# Novel Rodent Coronavirus-like Virus Detected Among Beef Cattle with Respiratory Disease in Mexico

**DOI:** 10.3390/v17030433

**Published:** 2025-03-18

**Authors:** Ismaila Shittu, Judith U. Oguzie, Gustavo Hernández-Vidal, Gustavo Moreno-Degollado, Diego B. Silva, Lyudmyla V. Marushchak, Claudia M. Trujillo-Vargas, John A. Lednicky, Gregory C. Gray

**Affiliations:** 1 Division of Infectious Diseases, Department of Medicine, University of Texas Medical Branch, Galveston, TX 77555, USA; isshittu@utmb.edu (I.S.); juoguzie@utmb.edu (J.U.O.); baldemar24@outlook.com (D.B.S.); lymarush@utmb.edu (L.V.M.); cltrujil@utmb.edu (C.M.T.-V.); 2 Faculty of Veterinary Medicine, Universidad Autónoma de Nuevo León, Escobedo 66054, Nuevo León, Mexico; hv10003@hotmail.com (G.H.-V.); gustavo.morenodgl@uanl.edu.mx (G.M.-D.); 3 Department of Environmental and Global Health, College of Public Health and Health Professions, University of Florida, Gainesville, FL 32610, USA; jlednicky@phhp.ufl.edu; 4 Emerging Pathogens Institute, University of Florida, Gainesville, FL 32610, USA; 5 Department of Microbiology and Immunology, University of Texas Medical Branch, Galveston, TX 77555, USA; 6 Institute for Human Infections and Immunity, University of Texas Medical Branch, Galveston, TX 77555, USA; 7 Department of Global Health, School of Public and Population Health, University of Texas Medical Branch, Galveston, TX 77550, USA

**Keywords:** rodent coronavirus (alphacoronavirus), cattle, epidemiology, emerging diseases, spillover

## Abstract

In February 2024, while conducting surveillance for novel respiratory viruses, we studied four beef cattle farms near Monterrey, Mexico. Nasal swabs were collected from sick and healthy beef cattle along with 3 h aerosol samples. None of the samples had molecular evidence of influenza A viruses. Three (8%) of thirty-six nasal swabs collected from the four farms and four (33.3%) of the twelve bioaerosol specimens had molecular evidence of influenza D virus. Five sick cow nasal swabs and one bioaerosol sample on a single farm had molecular evidence of rodent coronavirus-like (RCoV), an alphacoronavirus. Three (60%) of the five RCoV-positive cattle nasal swabs also had molecular evidence of influenza D. Attempts to isolate the RCoV in Vero-E6, LLC-MK2, MDBK, and L-2 cells were unsuccessful. However, we were able to assemble ~60% of the RCoV genome using next-generation sequencing. The six RCoV-positive samples clustered with RCoV strains identified in China in 2021. During the last 12 months, we have studied an estimated 478 dairy and beef cattle nasal swabs on 11 farms in the US and Mexico, and these RCoV detections are the first we have encountered. While feed contamination cannot be ruled out, given the propensity of CoVs to jump species and that we detected RCoV only in the noses of sick cows on this one farm, we are concerned that these findings could represent an isolated RCoV spillover event. With this report, we are alerting veterinarians and cattle farm owners of our observations that RCoV may be a new cause of bovine respiratory disease.

## 1. Introduction

Pathogen spillover refers to the spread of microorganisms in different animal species, beyond the original hosts in which they are known to replicate and survive. The greater the number of hosts in which a particular organism can complete its life cycle, the greater its evolutionary advantages. During the last century, human and livestock populations have concomitantly grown rapidly, leading to increased opportunity for pathogens that reside in livestock to spill over to infect humans. When such spillover events become more frequent, they may eventually lead to human–human transmission, epidemics, and rarely to pandemics [1].

Among the animals most adapted to human environments are rodents [2]. Rodents have rapidly and efficiently adjusted to urban environments, reaching numbers that surpass human populations in the largest cities worldwide [3]. Rodents often flourish where food is abundant such as on livestock farms [4]. In large livestock farms, large populations of rodents have a higher probability of exchanging their pathogens with large populations of livestock. This continuous fluid exchange of pathogens constitutes a training ground for the genetic-driven adaptation of microorganisms [5]. Viruses in the *Coronaviridae* family are remarkable for their large RNA genomes and their tendency to generate novel strains that occasionally spill over to infect new species and cause epidemics [6,7,8]. The *Coronaviridae* family is classified into four genera: *Alphacoronavirus*, *Betacoronavirus*, *Gammacoronavirus*, and *Deltacoronavirus*. Viruses that belong to the genera *Alphacoronavirus* and *Betacoronavirus* typically infect both humans and animals, including rodents, while those of the genera *Gammacoronavirus* and *Deltacoronavirus* are associated with avian species but can also infect mammals. Among the Betacoronaviruses, *Middle East Respiratory Syndrome Coronavirus* (MERS-CoV), *Severe Acute Respiratory Syndrome Coronavirus* (SARS-CoV), and *Severe Acute Respiratory Syndrome Coronavirus 2* (SARS-CoV-2) have recently caused epidemics and pandemics [9,10]. Growing attention has been focused on coronaviruses since the emergence of SARS-CoV, MERS-CoV, and SARS-CoV-2 due to their public health significance. In conducting surveillance for novel respiratory viruses that may be associated with sick livestock, we studied nasal swabs from sick and healthy cattle, as well as aerosol samples collected from various US and Mexican farms.

## 2. Materials and Methods

### 2.1. Sample Collection

In February 2024, as part of an ongoing One Health livestock farm epidemiological study for novel respiratory viruses on livestock farms, we visited four beef cattle farms near Monterrey, Mexico. We collected 20 nasal swabs (in cases where the number of cattle on the farms was lesser, we adjusted our sampling accordingly) from cattle, with approximately 70% exhibiting respiratory symptoms, including fever and nasal discharges and four bioaerosols specimens from each farm. The nasal swabs and bioaerosol samples were collected on four farms following procedures described in our previous studies [11,12]. Briefly, nasal swabs were collected by veterinary staff using polyester swab sticks, placed in a viral transport medium (Rocky Mountain Biological LLC, Missoula, MT, USA), and transported on ice to the Faculty of Veterinary Medicine and Zootechnics, Autonomous University of Nuevo León (UANL), where they were preserved at −80 °C. Bioaerosol samples were collected using TE-BC251 NIOSH bioaerosol cyclone samplers (Tisch Environmental Inc., Cleves, OH, USA), which were run for ~3 h at a flow rate of 3.5 L per min as described previously [13]. After collection, the material collected in the two tubes of the cyclone samplers and their filters was flushed with 1 mL of 0.5% bovine serum albumin (ThermoFisher Scientific, Waltham, MA, USA) each and eluted into 2 mL microtubes, resulting in a total of twelve specimens from the four bioaerosol samplers. All specimens were cryopreserved at −80 °C at the UANL and were later transported in a frozen state to our laboratory at the University of Texas Medical Branch (UTMB) in Galveston, TX, USA, where they were screened for influenza and coronaviruses using a real-time RT-PCR and a conventional RT-PCR pan-species coronavirus (pan-CoV) assay.

### 2.2. Molecular Screening

Following the manufacturer’s instructions, we extracted RNA from material collected by the bioaerosol samplers and cattle nasal swabs using a QIAamp Viral RNA Mini Kit (Qiagen, Valencia, CA, USA) on an automated QiaCube extraction platform (Qiagen, Valencia, CA, USA). The extracted RNA was screened by qRT-PCR for influenza A [14] and influenza D [15] viruses and for coronaviruses using a pan-species conventional RT-PCR pan-CoV protocol [16]. The PCR amplicons were visualized on a 2% agarose gel, and the expected molecular size amplicons were submitted for Sanger sequencing.

### 2.3. Next-Generation Sequencing (NGS) and Bioinformatics

To further characterize the virus genomes that had been detected, we employed a metagenomic next-generation sequencing (mNGS) approach following established protocols [17,18]. Library preparation was performed using the Illumina Nextera XT kit, and sequencing was carried out on the Illumina NovaSeq X platform using the NovaSeq X Plus series PE150 kit (Illumina, San Diego, CA, USA).

Raw sequencing reads were analyzed using the Chan Zuckerberg ID metagenomic platform (https://czid.org/). Phylogenetic analysis was conducted by downloading representative rodent coronavirus (RCoV) sequences from the National Center for Biotechnology Information and performing multiple sequence alignments using Multiple Alignment using Fast Fourier Transform [19]. A midpoint-rooted maximum likelihood phylogenetic tree was subsequently constructed using IQ-TREE v2.3.6 [20] and visualized with FigTree v1.4.4 (http://tree.bio.ed.ac.uk/software/figtree/, accessed 18 February 2024).

### 2.4. Cell Culture

All cell culture work was carried out in a Biosafety Level 3 enhanced (BSL3E) laboratory at UTMB. The cells used for virus isolation were obtained from the American Type Culture Collection (ATCC, Manassas, VA, USA). To isolate the rodent coronavirus from the pan-CoV RT-PCR positive nasal and bioaerosol specimens, we inoculated monolayers of Vero-E6 (African green monkey kidney cells; ATCC; CRL-1586), LLC-MK2 (Rhesus monkey kidney cells; ATCC; CCl-7), MDBK (Madin–Darby bovine kidney; ATCC; CCL-22), and L2 (rat lung cells; ATCC; CCL-149). Briefly, Vero-E6, LLC-MK2, and MDBK cells were propagated in Dulbecco’s Modified Eagle Medium with L-glutamine (DMEM, ThermoFisher Scientific, Waltham, MA, USA, cat no. 11965092), whereas the L2 cells were cultured in Ham’s F-12K (Kaighn’s) medium (F-12K; ThermoFisher Scientific, cat no. 21127022). All growth media were supplemented with 10% fetal bovine serum (FBS, ThermoFisher Scientific cat no. 26140-079) and 1X penicillin–streptomycin (ThermoFisher Scientific cat no. 15140-122). Maintenance media were prepared consisting of DMEM or F-12K, 0.1% bovine serum albumin fraction V (7.5%; BSA; ThermoFisher Scientific, cat. no. 15260-037), 1X penicillin–streptomycin (ThermoFisher Scientific cat no. 15140-122), HEPES buffer (ThermoFisher Scientific, cat. no. 15630-080), and 1 mM sodium pyruvate (Sigma-Aldrich, Saint Louis, MO, USA, cat. no. S8636-100ML). For each type of maintenance medium, parallel batches were made, one with and the other without 2 µg/mL of tosyl phenylalanyl chloromethyl ketone-treated trypsin (TPCK-trypsin, Sigma-Aldrich, cat. no. 4352157-1KT).

The cells were washed with phosphate-buffered saline (PBS; Corning, cat no. 21-031-CV) when they reached 75–85% confluency in 6-well plates. Before the inoculation of the cells, 0.2 mL of pan-CoV RT-PCR positive nasal swabs and bioaerosol specimens were mixed with 0.8 mL of the maintenance medium and filtered using a 0.45 μm pore-size filter (Millipore Sigma™ Millex™-HV Sterile Syringe Filter Unit, PVDF, 0.45 μm, Millipore, Burlington, MA, USA, cat. no. SLHV033RS). The filtrates were then inoculated onto the cell monolayers, which were incubated at 37 °C in a 5% CO_2_ environment for 1 h to allow for adsorption. After incubation, 2 mL of maintenance medium was added to the cell monolayers, and they were incubated at 37 °C in a 5% CO_2_ environment. At 4–5 days post-infection (pi), the media were harvested from the 6-well plates, and the cells were replenished with 1 mL of fresh maintenance medium. The final harvesting of the media and cells was carried out after the cells were freeze-thawed 7 days pi.

Virus culture harvests were treated with TRIzol LS Reagent (Invitrogen, Waltham, MA, USA) in the BSL3E laboratory using BSL3E work practices. They were then moved to BSL2E for RNA extraction, following the manufacturer’s recommendations, and stored at −80 °C. To determine whether viruses had been recovered, the extracted RNAs were analyzed using pan-CoV RT-PCR [16].

## 3. Results

### 3.1. Molecular Screening

No specimens from the four farms had molecular evidence of influenza A. However, seven (19.4%) nasal swabs and four (33.3%) bioaerosol specimens had molecular evidence of influenza D with high cycle threshold (Ct) values (Table 1). Based on our previous experience, cultures for viruses from specimens with such high Ct values do not yield isolates. Hence, we did not attempt to culture the influenza D viruses. Additionally, eight (seven nasal swabs and one bioaerosol) specimens were positive according to the pan-CoV assay. Sanger sequencing of pan-CoV amplicons identified six (five sick cow nasal swabs and one bioaerosol sample) out of the eight pan-CoV-positive amplicons as rodent-like coronavirus (RCoV) (GenBank accession number for the six specimens: PV067190-PV067195), while the remaining two specimens were identified as having bovine coronavirus. Notably, three (60%) of these five infected cattle also had co-infections with influenza D virus (Table 1).

### 3.2. Next-Generation Sequencing (NGS) and Bioinformatics

We further sought to characterize the RCoV virus through NGS and were able to assemble about 60% of the genome following NGS data analysis. The genome region coverage includes the 5′ end of the open reading frame genes 1a and 1b, as well as the nucleocapsid gene at the 3′ end. Among the six detections, they were 99.8% similar. Examining the six RCoV-positive samples individually, the percentage homology between ORF1a and ORF1b ranged from 16.1% to 18.3%. Comparing the six RCoV-positive samples against each other, there were excellent identity scores for ORF1a (100%) and ORF1b (99.8%). The Basic Local Alignment Search Tool analysis of the partial genome showed 94% similarity to a rodent coronavirus sequence with a GenBank accession number OQ297716.1.

**Figure 1:**
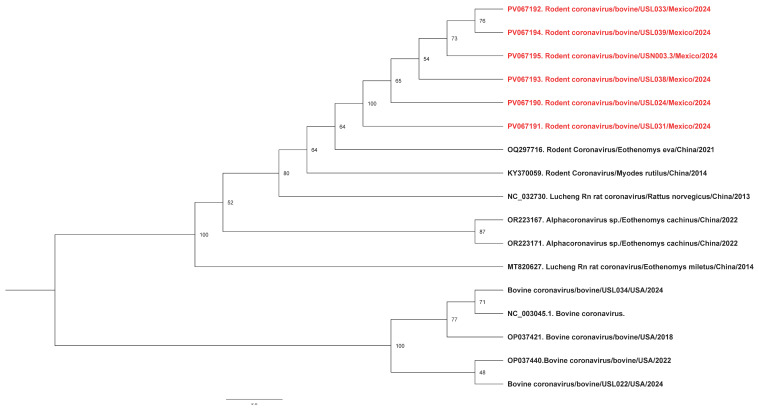
Phylogenetic analysis of rodent coronaviruses identified in this study. Maximum likelihood phylogenetic trees indicate that our sequences (highlighted in red) cluster together, suggesting a possible common source.

Phylogenetic analysis of the RNA-dependent RNA polymerase gene of the RCoV found it to cluster with the same rodent coronavirus OQ297716.1 previously identified in China in 2021 (Figure 1). In addition, the pairwise identity between RCoV and BCoV detections was 64.1%, suggesting they were very different viruses.

### 3.3. Cell Culture

We used four cell lines in attempting to isolate the RCoV, but after three blind passages per cell line, we failed to see cytopathic effects. In addition, the harvests from the cell cultures were negative according to the pan-CoV assay (Table 1).

## 4. Discussion

Since the beginning of this century, a number of novel coronaviruses (CoVs) have emerged to cause epizootics among humans [21]. The largest human epidemic was the pandemic caused by SARS-CoV-2, which, since 2019, has resulted in an estimated 776 million human illnesses and approximately 7 million deaths globally (as of December 2024) [22].

In previous active surveillance of spillover events, our research teams documented human influenza viruses and enteroviruses in pigs [23,24], influenza D virus among poultry [25], vampire bat-like enteroviruses among humans [26], among others. Recently, we isolated and cultured a novel canine coronavirus (CCoV-HuPn-2018) in nasopharyngeal swab samples from hospitalized pneumonia patients in Sarawak, Malaysia [27].

In this study, we found evidence of a very similar RCoV in the nasal swabs of five sick beef cows and one farm aerosol specimen in a single beef cattle farm near Monterrey, Mexico. To the best of our knowledge, this represents the first evidence of RCoV in sick cattle. It is important to note that RCoV, an alphacoronavirus, is distinct from bovine coronavirus (a betacoronavirus), which is commonly associated with respiratory infections in cattle. While this finding might be explained by rodent-contaminated feed, we did not find molecular evidence of this virus in the healthy cattle we studied from the same and other farms during this sampling period. Additionally, among more than 350 nasal swabs we collected from sick cattle on 11 other US and Mexican farms during the period March 2024 to January 2025, RCoV was only detected in the sick cows on this one farm. Hence, we hypothesize that the detected RCoV contributed to the respiratory illnesses in the studied cattle. Previous reports have shown the phylogenetic clustering of RCoVs with those from bats [28] and alphacoronaviruses from rodents with rabbits [29], suggesting cross-species transmission. The RCoV in this study grouped with RCoV sequences previously detected and reported in China [28], suggesting a common source. It has been shown that RCoVs have a common origin and only change over time via co-divergence with various hosts and interspecies transmission [30]. Additionally, we noted that 60% of the cattle nasal swabs with molecular evidence of an RCoV also had molecular evidence of influenza D virus. As far as we know, this is the first evidence of a co-infection involving both rodent coronavirus and influenza D.

These observations are concerning as they may represent a newly recognized CoV spillover event. Our study is limited because we could not obtain a virus isolate or assemble a complete genome for better insight into RCoV characteristics. We also did not have a way to examine cattle sera for evidence of infection with the RCoV. During the COVID-19 pandemic, caused by the SARS-CoV-2 virus, several studies reported evidence of coronaviruses including alphacoronaviruses in rodents, suggesting their exposure to these viruses [31,32,33].

Rodents are estimated to constitute more than 40% of all mammalian species, and some are known to carry zoonotic pathogens. However, until recently, there have been sparse studies of coronaviruses among rodents [34]. It seems prudent to conduct future surveillance for novel coronaviruses among sick livestock. If RCoVs or other novel viruses are prevalent among sick cattle, we need to confirm whether they are pathogenic and then better understand their epidemiology with the goal of preventing their transmission. In summary, our findings underscore the importance of surveillance for novel viruses at the animal–human interface as part of pandemic preparedness.

## Figures and Tables

**Table 1:** Descriptive data and molecular assay results from sick cattle with evidence of rodent coronavirus. Only farms 3 and 4 had molecular detections.

Sample ID	Sample Type	Animal Age (Months)	Symptoms	Quantitative RT-PCR (qRT-PCR) Result Influenza D (FluD)	qRT-PCR Cycle Threshold Values for FluD	Pan-CoV Assay Result (GenBank Accession)
Farm 3						
USL024-MX	Nasal swab	12	Nasal discharge and fever	Positive	35.44	RCoV (PV067190)
USL031-MX	Nasal swab	10	Nasal discharge and fever	Positive	35.36	RCoV (PV067191)
USL033-MX	Nasal swab	10	Nasal discharge and fever	Positive	31.14	RCoV (PV067192)
USL038-MX	Nasal swab	16	Nasal discharge and fever	Negative	>45	RCoV (PV067193)
USL039-MX	Nasal swab	17	Nasal discharge and fever	Negative	>45	RCoV (PV067194)
USN003.3-MX	Bioaerosol	Not applicable	Not applicable	Negative	>45	RCoV (PV067195)
Farm 4						
USL008-MX	Nasal swab	6	Nasal discharge	Positive	16.8	Negative
USL009-MX	Nasal swab	7	Nasal discharge and fever	Positive	16.74	Negative
USL010-MX	Nasal swab	6	Nasal discharge and depressed	Positive	33.8	Negative
USL011-MX	Nasal swab	8	Nasal discharge and fever	Positive	36.6	BCoV
USL019-MX	Nasal swab	6	Nasal discharge and fever	Negative	>45	BCoV

Pan-CoV = pancoronavirus assay; BCoV = bovine coronavirus.

## Data Availability

The original contributions presented in this study are included in the article. Further inquiries can be directed to the corresponding author.
